# Strengthening the Paediatricians Project 2: The effectiveness of a workshop to address the Priority Mental Health Disorders of adolescence in low-health related human resource countries

**DOI:** 10.1186/1447-056X-9-3

**Published:** 2010-02-18

**Authors:** Paul SS Russell, Muttathu KC Nair

**Affiliations:** 1 Child and Adolescent Psychiatry Unit, Department of Psychiatry, Christian Medical College, Vellore 632 002, Southern India, India; 2 Child Development Centre, Thiruvananthapuram Medical College, Thiruvananthapuram 695 011, Southern India, India

## Abstract

**Background:**

Paediatricians can be empowered to address the Priority Mental Health Disorders at primary care level. To evaluate the effectiveness of a collaborative workshop in enhancing the adolescent psychiatry knowledge among paediatricians.

**Methods:**

A 3-day, 27-hours workshop was held for paediatricians from different regions of India under the auspices of the National Adolescent Paediatric Task Force of the Indian Academy of Paediatrics. A 5-item pretest-posttest questionnaire was developed and administered at the beginning and end of the workshop to evaluate the participants' knowledge acquisition in adolescent psychiatry. Bivariate and multivariate analyses were performed on an intention-to-participate basis.

**Results:**

Forty-eight paediatricians completed the questionnaire. There was significant enhancement of the knowledge in understanding the phenomenology, identifying the psychopathology, diagnosing common mental disorder and selecting the psychotropic medication in the bivariate analysis. When the possible confounders of level of training in paediatrics and number of years spent as paediatrician were controlled, in addition to the above areas of adolescent psychiatry, the diagnostic ability involving multiple psychological concepts also gained significance. However, both in the bivariate and multivariate analyses, the ability to refer to appropriate psychotherapy remained unchanged after the workshop.

**Conclusions:**

This workshop was effective in enhancing the adolescent psychiatry knowledge of paediatricians. Such workshops could strengthen paediatricians in addressing the priority mental health disorders at the primary-care level in countries with low-human resource for health as advocated by the World Health Organization. However, it remains to be seen if this acquisition of adolescent psychiatry knowledge results in enhancing their adolescent psychiatry practice.

## Introduction

In most of the developing countries training in adolescent psychiatry are largely limited to mental health specialists; pediatricians, and other primary care physicians receive little training. As a result, effective diagnostic, treatment and prevention models that have been developed are not yet widely applied at the primary-care paediatric settings [1,2]. However, it is possible to train primary-care paediatricians to use standardized diagnostic criteria to screen psychiatric disorders in their clinical practice [3] and strengthen them to develop primary-care adolescent mental health services [4].

It has been suggested that gaining practical experience and training in adolescent psychiatry is an effective way for the paediatrician to acquire perspectives and skills that is helpful in hospital or community paediatrics [5]. Many models have been suggested to acquire knowledge and clinical skills by paediatricians, which include a training module in the undergraduate medical training and postgraduate training in paediatrics, integrating the child and adolescent psychiatry training program in to conferences, conducting special workshops and finally as Continuing Medical Education modules [6].

It is evident from the mental health burden and resources mismatch in developing countries that national efforts are required to restructure paediatric mental healthcare delivery [7]. Providing training to paediatricians in adolescent psychiatry, whose undergraduate and postgraduate training programs invariably excludes this discipline, will help tide over the resource paucity [7]. In India, National task Force on Family Life and Life Skill Education (NFLLSE) of the Adolescent Paediatrics Chapter of the Indian Academy of Paediatrics encourages training in adolescent mental health. As part of the Postgraduate Diploma in Adolescent Paediatrics by Child Development Centre and University of Kerala a three-day workshop was conducted to address the training needs of the paediatricians in identifying and treating the PMHD among adolescents.

Adolescent Psychiatry training in such special workshops may require evidence of their effectiveness, and this type of data has been difficult to obtain. In an earlier paper we have studied the need, content and process of *Strengthening the Paediatrician Project*, a collaborative workshop for paediatricians on adolescent psychiatry with psychiatrists. Here we focus on the effectiveness of the workshop in enhancing adolescent psychiatry knowledge among paediatricians.

## Methods

We review only the relevant aspects of our methods here; an extensive description of the workshop has been presented in the accompanying paper [6] and details are reported using the Consolidated criteria for Reporting Qualitative Research (COREQ) guidelines.

### Setting and participants

Data used in the present analyses are from the *Strengthening the Paediatrician Project *(SAPP), a seminal adolescent psychiatry workshop to explore the need, content, process and effectiveness of a workshop for paediatricians in collaboration with psychiatrists. The workshop was conducted at the Child Development Centre, Thiruvananthapuram from the 19^th ^to 21^st ^of June 2006 under the auspices of the NFLLSE of the Indian Academy of Paediatrics was for 27 hours and spread over three days.

### Interventional Workshop

In brief, the *content facilitation *was done by psychiatrists (facilitators) who were proficient in the theory and practice of psychiatry. The participants were divided in to five groups of about 8-10 each and five psychiatrists invited by the NFLLSE facilitated each group. After the pre-workshop assessment of knowledge related to adolescent psychiatry, the two morning sessions in the *first day *introduced the Adolescent Psychiatry as well as systems in mind with their phenomenon and related psychopathology identifiable in a mental status examination. The two sessions in the afternoon focused on identifying the psychopathology with case vignettes and interviewing 'cases' (enacted by psychiatrists) for the psychopathology. The morning session of the *second day *translated the psychopathology noted in the mental status examination of the 'cases' to the *International Classification of Diseases: Mental and Behavioral Disorders *(Clinical Descriptions and Diagnostic Guidelines) - Tenth version (ICD-10) [8] based diagnosis of priority mental health disorders that paediatricians will encounter. The afternoon session deliberated on PHMD that needs non-pharmacological interventions and the non-pharmacological interventions. The forenoon session of the *third day *addressed the PMHD that requires medication and pharmacotherapy of these disorders. In the post-lunch session, the post-workshop assessment was conducted.

The *process facilitation *included didactic teaching using audio-visual materials, interacting with the child psychiatrist to clarify their queries, small group case-work up with case vignettes, presenting the case by a participant from each group supported by the respective group member, identifying the psychopathology and diagnosing the PMHD using role plays, conducting diagnostic interviews of the 'cases' based on ICD-10 diagnosis. Finally of the analysing of the video recording of the interviews by the members of each group and then along with their facilitators as well as the child psychiatrist for interviewing skills, mental status examination and diagnostic formulation was conducted.

### Measure

A brief questionnaire to assess the acquisition of adolescent psychiatry knowledge by pediatricians was specifically developed and used. It had five multiple choice items and each correct endorsement was given a score of one and thus a score range of 0-5 was possible. Each of the five items was intended to evaluate one of the five areas namely: (1) understanding of the phenomenology, (2) identification of the psychopathology, (3) diagnosis of the PMHD, (4) selection of the appropriate psychotropic medication and (5) non-pharmacological interventions for which appropriate referrals will have to be made. The same multiple-choice questions were completed at the beginning of Day 1 before the first session began and during the last session of Day 3 before the focused group discussion.

### Data analysis

The descriptive data was presented in percentages and the acquisition of knowledge was analysed using Wilcoxon Matched-Pairs Signed-Ranks test. Multivariate linear regression was done to adjust for the confounding effect of the level of paediatric training, and years of experience in paediatric care. To account for the 14.6% of the participants who left the workshop immediately prior to the closing session to catch commuter and intercity trains we did an intention- to- participate analysis where the pretest scores brought forward as the posttest scores for analysis. Significance was set at P < 0.05 (two tailed) and data was analysed using SPSS 11.5.

## Results

The participant characteristics are described in the accompanying paper [6].

### Acquisition of knowledge

In the bivariate analysis, the overall knowledge of the paediatricians statistically significantly improved after the workshop. The maximum gain was in the areas of understanding the phenomenology, identifying the psychopathology and diagnosis of common mental disorder as well as selecting the psychotropic medication. The areas that did not show statistically significant gains were making diagnoses that involved multiple psychological constructs (like the levels of psychological conflict, topography of mind required to differentiate malingering disorder, factitious disorder and dissociative disorder) and ability to refer for appropriate psychotherapy (Table 1).

**Table 1:** The change in child and adolescent psychiatry knowledge of paediatricians following the three day workshop.

*Item*	*Pre workshop Mean (sd)*	*Post workshop Mean (sd)*	*Unadjusted difference Z value*	*P Value*	*Adjusted difference^a ^β(SE) value*	*P Value*
1. When an adolescent says that he has been hearing people talk bad things about him when no one is talking about him, when he is fully awake and conscious. What is this phenomenology?	0.40(0.49)	0.57(0.50)	-2.5	0.01	1.0(0.27)	0.001
2. When an adolescent complaints to you about racing of thoughts, what psychiatric disorder is this psychopathology suggestive of?	0.37(0.49)	0.53(0.50)	-2.9	0.004	1.6(0.32)	0.001
3. What is your medication of choice for depression among adolescents?	0.28(0.45)	0.52(0.50)	-3.5	0.001	1.4(0.28)	0.001
4. When an adolescent girl comes to you with weakness of her right hand and all the lab investigations are normal and has significant stressful life events, what is her possible psychiatric diagnosis?	0.10(0.30)	0.20(0.40)	-1.7	0.08	1.3(0.29)	0.001
5. What form of psychotherapy is cognitive therapy?	0.18(0.39)	0.22(0.41)	-5.3	0.5	0.17(0.32)	0.5
Total score	1.33(1.44)	2.0(1.7)	-4.0	0.001	1.7(0.36)	0.001

^a ^= adjusted for level of training in paediatrics and number of years spent as paediatrician

In the multivariate regression analysis when the possible confounders namely the level of training in paediatrics and the number of years of experience in treating children were adjusted, the acquisition in overall knowledge, understanding of phenomenology, identifying psychopathology, diagnosing the PMHD and selecting the psychotropic remained significant as in the bivariate analysis (Table 1). However, the ability to diagnose disorders involving multiple psychological concepts gained significance, as the years of experience also significantly confounded the ability to diagnose disorders with multiple psychological concepts (P = 0.05). The years of experience the paediatrician had also significantly confounded the ability to understand phenomenology (P = 0.006). The level of paediatric training did not significantly confound any area in the acquisition of knowledge among the participants. Despite controlling for the confounders the ability to refer the adolescents for appropriate psychotherapy did not show any significance improvement.

## Discussions

We are not aware of any study documenting the effectiveness of an inter-disciplinary collaborative workshop to enhance the acquisition of adolescent psychiatry knowledge among academic and practicing paediatricians using multimodal training techniques. However, the findings from our study are consistent with that of other studies found in the pharmacy, nursing and medical education literature.

### Effectiveness of the workshop

Our findings suggest that this collaborative, multimodal training approach to teaching is enjoyable and effective in the acquisition of theory and clinical skills related to adolescent psychiatry. The authors speculate that paediatricians possibly displayed a positive attitude to learn adolescent psychiatry even before attending the training workshop. However, they acquired adolescent psychiatry knowledge and skill significantly after the workshop. A statistically significant increase in questionnaire responses was observed in 4 of the 5 of the questions and therefore, it can be concluded that there was a relationship between the training intervention and the increase in knowledge for all but one area.

When the mental illnesses addressed in the workshop were viewed from a biopsychosocial perspective, the significant increase in knowledge of phenomenology, diagnosis and pharmacological management (medical constructs of mental illnesses) and insignificant improvement of knowledge in psychological management and referral for psychotherapy (psychosocial constructs of mental illnesses) suggest that the workshop was more successful at increasing 'medical constructs' and less successful at changing 'psychosocial constructs' of the paediatricians relevant to adolescent psychiatry.

### Recommendations to paediatric education

Research on the outcomes of educational improvement interventions can be utilized to strengthen the theoretical basis for required regulatory training as well as to validate interventions for health-care education. This knowledge and skill acquisition suggests that when this adolescent psychiatry module is added in the various training processes like postgraduate training or CME successfully increase the knowledge towards the identification of psychopathology, a classificatory system based diagnosis of disorders, psychopharmacological management and feasible psychological interventions or referrals as recommended by World Health Organization [7]. Also, continual assessment of participants' knowledge with such learning experience will occur so that we can incorporate this into appropriate areas of the paediatric training. Other potential uses for this multimodal training tool are in assessing participants' communication skills, either with the patient, family members or other health care professionals. Also, this workshop may provide continuing education opportunities for senior paediatric faculty responding to policy needs in institutional settings.

### Recommendations for paediatric practice

The workshop elements focused strongly on cognitive knowledge, with the assumption that an increase in knowledge would result in a concomitant improvement of attitudes, and practicing skills. It may be possible to develop an additional training element that specifically addresses underlying assumptions and fears that can compromise the clinical skills that should emerge from the knowledge gained. Such a training workshop might utilize open discussions or hands-on approaches. The addition of a clinical psychologist to the multidisciplinary training team may improve the outcome.

### Recommendations for future research

Further research is needed to focus on the specific components of the workshop. While this study evaluated the impact of the entire multi-element workshop, no conclusions could be drawn for the individual elements of the intervention such as the didactic sessions, case-vignettes, simulated case workups, or video feedbacks. Which of these intervention elements had the most impact on increasing the knowledge is conjectural. The study should also be extended to other teaching settings (like conference and CME programs) and the teaching elements themselves could then be modified to include other methods designed to specifically address these settings (real cases in CME programs). In the future, based on the positive response, this multimodal training with a collaborative approach will be continued in the Postgraduate Diploma in Adolescent Paediatric health training program at Child Development Centre. The next learning experience will occur in our Postgraduate Diploma in Developmental Neurology program in which neurologist will learn basic theory and practice of adolescent psychiatry.

### Limitations

The main caveats of this study are the specific nature of the training subject and the nature of the population. Firstly, the study assumed that the choice of data gathering instruments was appropriate for the task at hand. While the present study utilized a specific set of knowledge evaluation questions that concentrated on what the interdisciplinary team believed represented appropriate concerns of paediatricians facing adolescent mental health issues at the primary care level, all of the specific needs of the paediatricians at different practice settings were not assessed during this study. An expanded and validated knowledge evaluation instrument could be beneficial in identifying real knowledge acquisition. Secondly, this study teases out the adolescent psychiatry component of a multicomponent workshop for measurement and therefore lack of a comprehensive measure inclusive of the various components of the workshop could have negatively affected the performance of the participant in answering the questionnaire. Finally, as this study utilized voluntary participation rather than specific random sampling, extensions of these conclusions to other paediatricians are understandably weakened as possibly paediatricians motivated to learn the discipline of adolescent psychiatry only responded.

In conclusion, this model of inter-disciplinary collaborative, multimodal educational workshop is effective in enhancing the adolescent psychiatry knowledge among paediatricians. However, it remains to be seen if the paediatricians are able to retain the acquired knowledge of adolescent psychiatry and apply in their clinical practice as well. If such information retention and application follows, this model of strengthening the paediatricians can partly help reinforce the efforts of WHO in addressing the Priority Mental Health Disorders among the adolescents. Further studies to explore if the acquired adolescent psychiatry knowledge is applied and thus integrated in clinical practice are required.

## List of abbreviations

CME: Continuing the Medical Education; ICD-10: International Classification of Diseases: Mental and Behavioral Disorders (Clinical Descriptions and Diagnostic Guidelines) - Tenth version; NFLLSE: National task Force on Family Life and Life Skill Education; PMHD: Priority Mental Health Disorders; SAPP: Strengthening the Paediatrician Project.

## Competing interests

The authors declare that they have no competing interests.

## Authors' contributions

PSSR was involved in the conception, designing, data analysis and interpretation, drafting and approving the final version. NMKC was involved in the conception, drafting and revising the final draft. All authors read and approved the final manuscript.
